# Transition of care: experiences and preferences of patients across the primary/secondary interface – a qualitative study

**DOI:** 10.1186/1472-6963-9-62

**Published:** 2009-04-07

**Authors:** Annette J Berendsen, G Majella de Jong, Betty Meyboom-de Jong, Janny H Dekker, Jan Schuling

**Affiliations:** 1 Department of General Practice, University Medical Centre Groningen, University of Groningen, Ant. Deusinglaan 1, 9713 AV Groningen, the Netherlands

## Abstract

**Background:**

Coordination between care providers of different disciplines is essential to improve the quality of care, in particular for patients with chronic diseases. The way in which general practitioners (GP's) and medical specialists interact has important implications for any healthcare system in which the GP plays the role of gatekeeper to specialist care. Patient experiences and preferences have proven to be increasingly important in discussing healthcare policy. The Dutch government initiated the development of a special website with information for patients on performance indicators of hospitals as well as information on illness or treatment.

In the present study we focus on the transition of care at the primary – secondary interface with reference to the impact of patients' ability to make choices about their secondary care providers. The purpose of this study is to (a) explore experiences and preferences of patients regarding the transition between primary and secondary care, (b) study informational resources on illness/treatment desired by patients and (c) determine how information supplied could make it easier for the patient to choose between different options for care (hospital or specialist).

**Methods:**

We conducted a qualitative study using semi-structured focus group interviews among 71 patients referred for various indications in the north and west of The Netherlands.

**Results:**

Patients find it important that they do not have to wait, that they are taken seriously, and receive adequate and individually relevant information. A lack of continuity from secondary to primary care was experienced. The patient's desire for free choice of type of care did not arise in any of the focus groups.

**Conclusion:**

Hospital discharge information needs to be improved. The interval between discharge from specialist care and the report of the specialist to the GP might be a suitable performance indicator in healthcare. Patients want to receive information, tailored to their own situation. The need for information, however, is quite variable. Patients do not feel strongly about self-chosen healthcare, contrary to what administrators presently believe.

## Background

Coordination between care providers of different disciplines is essential to improve the quality of care, in particular for patients with chronic diseases [1]. These patients are often treated by different health care providers simultaneously. The way in which general practitioners (GP's) and medical specialists interact has important implications for any healthcare system in which the GP plays the role of gatekeeper to specialist care (the Netherlands and the UK). However, in countries without a gatekeeper system, coordination of care is a concern as well [2]. A large European study showed that patients in different countries value different aspects of healthcare [3]. There are, however, similarities in the ranking order. 'Being taken seriously' is generally regarded as most important and 'waiting time in the general practitioner's office' least important.

When evaluating the transition from primary to secondary care and vice versa, we need to have a thorough knowledge of the experiences and preferences of patients. Qualitative and quantitative research regarding these issues have been done in the United Kingdom [4,5]. Five themes emerged. The first four were: "getting in" (access to appropriate care), "fitting in" (orientation of care to the patient's requirements), "knowing what's going on" (provision of information), and "continuity" (continuity of staff and coordination and communication among professionals). The fifth theme was "limbo" (difficulty in making progress through the system), which was influenced by failures in care in relation to the other four themes. Three types of continuity were identified: Informational, Management, and Relational continuity [6].

Other research has focused on patients suffering from specific conditions [7,8] or patients' experience with a single part of the transition between primary and secondary care, such as the referral [9], hospital stay and discharge [10-12], terminating specialist care [13], patients' experience with a new form of cooperative care [14], and chronically ill patients [15,16].

Patient experiences and preferences have proven to be increasingly important in discussing healthcare policy. This trend follows a client-centred perspective, which allows patients to choose the type of care they wish to receive in relation to the referral [17,18]. The Dutch government initiated the development of a special website (portal: KiesBeter.nl) with information for patients on performance indicators of hospitals as well as information on illness or treatment. Patient experience will be an important indicator of the quality of care [19].

In the present study we focus on the transition of care at the primary – secondary interface. We developed the following questions.

(a) What are the experiences and preferences of patients regarding the transition between primary and secondary care?

(b) What information do patients wish to have on illness/treatment and how should the information be provided?

(c) Is having a free choice of secondary care provider (choice of hospital or specialist) important to patients?

## Methods

We chose an explorative qualitative design with focus groups of patients with varying conditions referred to specialist care.

### Patient groups

We selected from the two databases of the registration networks of the General Practice Departments at the Universities of Groningen and Leiden (three single and six group practices; 60,000 patients) a random sample of patients with the following characteristics: during the past two years they had been referred to a specialist, they were older than 18 and they spoke Dutch. Patients referred to psychiatrists and patients who had had a stroke were excluded from the study.

We formed three groups of patients: chronic groups, major treatment groups and MUPS groups (Medically Unexplained Physical Symptoms). We expected important differences between these groups with regard to their experiences with the transition of care. The chronic groups consisted of patients with a chronic condition such as diabetes, COPD, rheumatic disease, multiple sclerosis and cardiac conditions. The major treatment groups consisted of patients who had suffered a coronary, a hip fracture, or pneumonia. The MUPS groups consisted of patients with symptoms, such as headache, stomach ache, palpitations, spastic colon, and fibromyalgia. We invited a total number of 330 patients by mail, of whom 89 responded that they were willing to participate. Eventually 71 patients participated in the study (4–9 participants per group): Five chronic groups (n = 31), four major treatment groups (n = 21), and four MUPS groups (n = 19). Meetings lasted approximately 90 minutes (80–100 minutes) and took place between November 2004 and August 2005.

### Collection of data

The focus group meetings took place at a neutral location in the cities of Groningen, Hoogeveen, and Leiden. The discussion was led by an experienced independent moderator, using a checklist with open questions derived from the main study questions:

• What are your experiences in the path from primary to secondary care and vice versa? Can you recommend improvements, even if you are satisfied?

• Were you provided with enough information? Who should give the information and how can this be improved?

• Do you want to make choices by yourself concerning the hospital, the specialist, or the form of treatment? If so, what enabling information is needed?

• Who should support you and your family?

In the chronic groups we also asked patients about their preferences with respect to ongoing care. Patients who had been hospitalized were asked about their experiences.

An employee from a local patient organization functioned as an observer and made notes.

The study design was reviewed by the Medical Ethics Committee of the University Medical Centre Groningen. This committee decided that the study did not require legal assessment. The participants did give permission for the audio recordings.

### Data analysis

The focus group discussions were recorded on tape and transcribed verbatim. The analysis was conducted according to the rules of qualitative research and the framework method [20,21]. The five most important steps were: familiarization, identifying a thematic framework, indexing, charting, and interpretation. Two researchers and a medical student independently labelled the transcripts. Any discrepancies were discussed until consensus was reached. The coding system was refined until no further codes were required. After discussion with other team members, codes were categorized and a coding scheme was devised. The interviews and the analyses were conducted simultaneously. The data were processed with the computer program Kwalitan 5.0 [22]. We did not perform an iterative process with the participants (member checking). We thought it too strenuous and time consuming for the patients. However, we checked our findings with the independent observer from the patient organization [23].

## Results

The average age of the 71 participants (37 men and 34 women) was 59 years with a range from 34 to 83 years. Eighty percent lived with a partner and 20% were single. The level of education in the group varied: primary school (4), GCSE (22), A-levels (25), and higher education/university (19).

Saturation occurred during the eighth focus group as was evidenced after the analysis of ten groups. The remaining three meetings were used to investigate trends in Leiden. There were no major differences between the participants in terms of age, gender or practice type.

**Figure 1:**
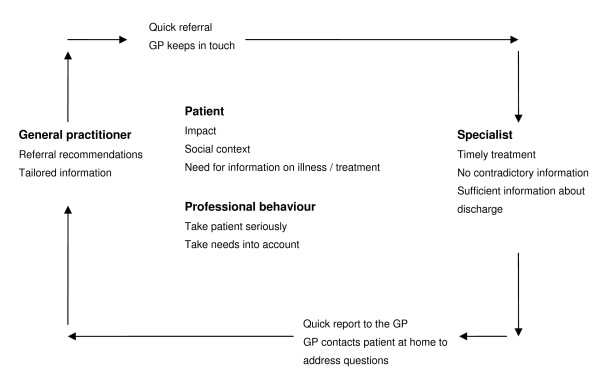
**Transition between primary and secondary care**.

The themes and their interrelations are presented in figure 1. The desire for free choice of type of care (hospital/specialist) will be discussed in the section on the referral.

### Impact on patients and their environment

#### Impact – experience

Many patients felt that their illness and a referral to the hospital influenced their cognitive functioning and their behaviour. The information provided by the specialist is often forgotten due to stress or pain.

A large number of patients stated that one needs to be quite assertive in the hospital setting e.g. in addressing waiting times, when the after care is deficient, when asking for test results and when receiving explanations of diagnostic tests.

"You're pretty sick, and though one person may crawl into the bushes like a sick animal, another may become aggressive, and that's more like me".

"I often try to see the bright side of things, but you are always somewhat tense. It is about your own body, after all, and if you are tense, you might not hear everything you should be hearing".

#### Social context

Many patients considered it important that attention is given to their caregivers, e.g. being provided information about handling the disease, being offered emotional support, and having their stress tolerance assessed. At the time of discharge, these concerns require special attention.

### Transition between primary and secondary care – GP

#### The referral

Almost all patients found it important that the GP referred them quickly and appropriately. They appreciated it when the GP was able to shorten the time span between the referral and the first consultation with the specialist, and when their GP stayed in contact with them after the referral.

#### Patient's choice

The need to make one's own healthcare choices did not spontaneously arise during the focus group sessions. When asked directly, many patients said they found it either too difficult or felt too ill to make their own choices.

Most of the patients said the GP plays an important role in making decisions with respect to the referral. A large number of subjects left their choices up to the GP entirely. Others prefer their GPs to inform them, advise them and consult with them, but to let them make their own decisions in the end.

"In such a case, you assume the GP has assessed what needs to be done and what hospital would be best for the job. You don't have a choice then, you simply can't make the decision, particularly not if you are mentally not competent, then you are dependent".

"Of course you can judge the hospital, but how can you judge the specialist? You can't put a label on someone and say that one is bad: ten of his patients have died".

#### Back to the GP

With the exception of patients who had undergone a minor procedure patients value being contacted by the GP after seeing a specialist or after hospital admission, to discuss any problems or further treatment. This could be accomplished with a telephone call, a visit, or by asking the patient to come into the office.

"When I don't understand something, I tell the specialist: hang on a minute, I'm a lay person, would you please explain everything to my GP and then he'll explain it to me in a language I can understand".

### Transition between primary and secondary care – specialist

#### Waiting times

Many patients reported having negative experiences with long waiting times for diagnostic investigations or for procedures and with long waits in waiting rooms. Patients who were seen quickly were positive.

"Dr. S. referred me to a colleague, but I returned to the GP, where I was seen the next day, but now I have to wait another three months before I can see the next specialist".

"Well, it was good, because you phone for an appointment, enter the code number, and you're given a date, please come at 10:00, and then all the tests are completed one after the other".

#### Making decisions with the specialist

Many patients felt that they had few options for care to choose from when consulting with the specialist.

#### Reporting back to the GP

Aconsiderable number of patients was dissatisfied with the length of time it took for the GP to be given word from the specialist; this could often take several months. Some subjects were also displeased with the contents of the report, they had the impression that the information the GP eventually received was incomplete. When the patient was under a specialist's care for a longer period of time, the GP often did not receive any notification in between. Furthermore, patients reported that they felt insecure when they were suddenly discharged from the specialist's care with no clear referral back to the GP.

On the other hand, a few patients were quite satisfied with the communication between their GP and the specialist. All patients felt however, that improvement is needed for the communication between GP and specialist. Information needs to be conveyed more rapidly, more completely, and, for the patient, the information should be more accessible.

### Professional behaviour

All patients found it important to be taken seriously, both by the GP and the specialist. Physicians should listen well to the patient, provide correct information and resources and inform the patient on the pros and cons of treatments and referrals. They should also make it possible for the patient to participate in the decision making process.

The subjects preferred the GP to actively maintain contact and, when necessary, to initiate contact. Almost all of the patients were satisfied with the GP's attitude, especially the patients in the chronic group. That is why most of the patients in the chronic group express a preference for follow-up with their GP.

A number of patients felt that the specialist did not sufficiently take the patient's individual needs into account. Many indicated the need to express their emotions during an admission, but said they had the impression that the specialist tended to avoid empathetic, supportive contact. On this criterion, subjects were generally more positive about GPs than about specialists, particularly patients in the MUPS group.

### Information

#### Need for information

The need for specific information expressed by each patient varied greatly. A small number of patients only wanted to hear practical information, whereas a larger number of patients wanted to receive more specific information about their prognosis. A few patients wanted to know every detail.

Many patients found that knowing everything makes matters worse. Many also found the contents of package leaflets for medication quite suggestive and frightening.

"I don't know if I need more information. It's not good for me. It may just increase my fear; make me more anxious".

"I'm the kind of person who wants to know everything, and I also want to observe my own operation".

"But then you read something, and you think to yourself: oohhhh. Will I get that? If you look at the enclosed leaflet, you think: tomorrow I'm history. Everything you can possibly get is included. I think you have to be critical when reading stuff like that".

#### Discharge from the hospital

All patients appreciated receiving complete information about discharge (time of discharge, reasons for discharge, review of admission). Such information was absent in a number of cases. Subjects also expressed the need for reassurance, which could be given through:

* information on daily rules and instructions concerning continuing treatment and the symptoms patients can expect;

* clear information on further policy and follow-up appointments at the clinic;

* concern and guidance, if necessary provided by a nurse, a medical social worker, or a physiotherapist. A number of patients would like the opportunity to consult by telephone after discharge.

Many patients found that communication was inadequate concerning follow-up, sources of information and to whom questions should be directed.

Quite a few patients were dissatisfied by the follow-up care they received from the specialist. This was most evident in the major treatment group.

"You've been discharged, and it's just like losing your job: you don't need to go back, because you've received your last pay cheque".

"Since there are pamphlets and information booklets about certain diseases anyway, you might as well make booklets about complications, recommendations, and practical post-surgery tips, for certain treatments".

#### Sources of information

##### *GP *

Many patients preferred receiving information from the GP. GPs tend to spend the necessary time and, as opposed to specialists, give more extensive information tailored to the patient. A few of the patients reported that the specialist was sometimes able to provide better information than the GP.

##### Specialist

All the patients valued receiving a clear diagnosis and to be informed in advance of what they could expect with a specific test, procedure, or treatment. Patients preferred to be informed beforehand if they would be seeing a different physician. A number of patients indicated that they had received inadequate information about hospital procedures.

Quite a few subjects were dissatisfied with the amount of information they were given about their condition, medication, treatment, or the consequences of an operation. Subjects had the impression that specialists did not have or take enough time for discussing the patient's concerns. Another, smaller, group of patients was quite satisfied with the information it received.

"If you want to speak with a doctor, you just have to put a stick in front of his door so that he trips over it, to put it bluntly".

"But a surgeon could also tell you: this is where you can obtain additional information, on the internet for example".

A number of subjects noticed there was poor communication among specialists. Receiving conflicting information from specialists and laboratory personnel was seen as a major problem. Patients did report, however, that they liked hearing results from the latter if that meant that they would hear the results as soon as the test was completed (ultrasound, x-ray).

Most of the dissatisfied subjects were members of the chronic and MUPS groups, whereas the satisfied patients were from the major treatment group.

"I hear three, four explanations in one day telling me what might be wrong with me. In the end you get angry, and ask to see the real doctor, because you finally want to know what's actually wrong with you".

##### Nursing staff

Many patients liked receiving information from nurses during their hospital stay or at the clinic. Reasons for this included clarity of the instructions, more extensive information, easy access to information and the thoroughness of nurses. Nurses can also help in communicating with the specialist. Most patients prefer the specialist to announce a diagnosis or change in treatment however.

##### Patient organization

Some patients do not believe in being involved with patient organizations and experience contact with people facing a similar medical condition as something involving a lot of whining and carrying on. Besides this, the meetings are often held too far away. These issues were raised primarily by patients who were not fully aware of the existence of patient organizations or who had minimal experience with them.

Others reported that it was good to hear anecdotes from other patients, as well as gaining tips which they could use to their advantage. Many patients also valued such organizations because of the extensive literature available regarding issues involving the disease (medical developments, self-management), but also regarding such practical things as organizing a mortgage. Patient organizations were mentioned most often in the chronic groups.

##### The pharmacy

A few patients from the chronic group thought pharmacies should provide information. The ones who had had experience with this were quite positive.

##### Miscellaneous

Many patients liked receiving extra literature so that they would have the chance to review information once they had returned home. Besides books, magazines, television programs, and medical encyclopaedias, the Internet was reported to be an important source of information. It was interesting to note that the age of the people who directly or indirectly consulted the Internet ranged from 34 to 79 years, with two thirds older than 50. The older patients often received Internet information through family members. Noteworthy is that these were better educated patients.

## Discussion

### Strengths and limitations

The strength of this study is that patients were able to speak freely supported by the presence of the independent observer from the patient organization. Research on how much information patients wish to receive and the importance of free choice for patients is new.

Since this is a qualitative study, the results have to be regarded as inductive. This study was conducted in the Netherlands, where the GP functions as a gatekeeper for specialist care. In countries with free access to specialist care preferences and experiences of patients might differ. Patients referred to psychiatrists and patients who had had a stroke were excluded. The first category of patients might need a different approach, while the second category was excluded because of their communicative impairments.

Each patient-doctor contact is a combination of communication and medical content. Patients' opinions as reported in our study are affected by the appreciation of the communicative skills of the doctors. We cannot discriminate between these aspects.

### Experiences and preferences

Patients want to be taken seriously and want their needs to be taken into account [3].

Most of the patients in the chronic groups express a preference for follow-up with their GP. Patients value the coordinating role of the GP, perhaps because of the relational continuity [6,9,24]. Chronically ill patients find empathy, provision of information, allotted time, and continuity of care the most important aspects while they are being monitored [15].

Patients place considerable value on progress through the system (an accurate and timely referral; to be seen quickly by a specialist), as shown in other studies [4,8,16]. Lack of progress was called earlier 'left in limbo' [4].

Many patients find that the specialist's report to the GP needs to arrive more quickly. Other studies have shown that patients find the quality of communication between the GP and the specialist important [7,8,13,16].

With regard to empathetic, supportive contact subjects were generally more positive about GPs than about specialists, particularly patients in the MUPS groups. Having no explanation for the physical symptoms of these patients, specialists may avoid contact out of embarrassment.

Being referred has an impact on the way a patient functions. Many patients state it is important to be sufficiently assertive.

In our study patients prefer to receive information from their GP, tailored to their particular situation, and presented in an understandable format without contradictions [4]. This preference is possibly influenced by the relational continuity the GP offers.

Patients from the chronic groups thought patient organizations and pharmacies could provide information. This is possibly due to the fact that many chronic patients visit the pharmacy regularly. Other research has shown that patients value information from different sources [7].

Receiving conflicting information from specialists and laboratory personnel was seen as a major problem. Most of the dissatisfied subjects were members of the chronic and MUPS groups, whereas the satisfied patients were from the major treatment group. Possibly, the first two groups meet different interpretations more frequently. Though patient perceptions of inter-professional communication may not accurately reflect the true state of affairs, patients state that the specialist's report to the GP should include more information.

Patients wish to be consulted about the timing of discharge and they wish improved information about what they can expect after discharge. They did not receive sufficient information about symptoms to be aware of and when to resume daily activities [11,13,25].

There was considerable variation in how much information is desired by individual patients. Some patients report that too much information increases their anxiety.

Our research shows that the GP plays an important advisory role during the referral (choice of hospital/specialist). This has been seen previously in research done in Germany, where the GP does not play the role of 'gatekeeper' [26]. Patients do not always wish to choose the type of care themselves, nor do they always feel capable of doing so [27]. On the other hand, with the specialist, patients do not always get the opportunity to make their own decisions.

## Conclusion

We formulated the following hypotheses:

• There is a serious lack of information addressing the problems the patient may face after discharge.

• The time that elapses before the specialist has reported back to the GP was generally found to be too long. This interval could function as a performance indicator.

• Information should be tailored to the patient's wishes; care providers should reckon with different types of information seekers and establish beforehand to which group their patient belongs.

• Patients desire room to discuss their pros and cons of options of care and expect guidance from their doctor; aiming for free choice in healthcare for all patients is an unrealistic approach. Contrary to the assumptions made by governmental bodies, many patients do not feel strongly about self-chosen healthcare.

Based on the results of this study a questionnaire will be developed. With the use of such an instrument it will be possible to measure patient opinions across the interface between primary and secondary care.

## Competing interests

The authors declare that they have no competing interests.

## Authors' contributions

All authors contributed to the design and the write-up of this study. AJB was responsible for the day-to-day management and produced the first draft of the manuscript. AJB and GMdJ analyzed the interviews together with JS. All authors read and approved the final manuscript.

## Pre-publication history

The pre-publication history for this paper can be accessed here:



## References

[B1] Haggerty JL, Reid RJ, Freeman GK, Starfield BH, Adair CE, McKendry R (2003). Continuity of care: a multidisciplinary review. BMJ.

[B2] Schoen C, Osborn R, Doty MM, Bishop M, Peugh J, Murukutla N (2007). Toward higher-performance health systems: adults' health care experiences in seven countries, 2007. Health Affairs (Millwood).

[B3] Groenewegen PP, Kerssens JJ, Sixma HJ, Eijk I van der, Boerma WG (2005). What is important in evaluating health care quality? An international comparison of user views. BMC Health Serv Res.

[B4] Preston C, Cheater F, Baker R, Hearnshaw H (1999). Left in limbo: patients' views on care across the primary/secondary interface. Qual Health Care.

[B5] Baker R, Preston C, Cheater F, Hearnshaw H (1999). Measuring patients' attitudes to care across the primary/secondary interface: the development of the patient career diary. Qual Health Care.

[B6] Freeman GK, Woloshynowych M, Baker R, Boulton M, Guthrie B, Car J, Haggerty JL, Tarrant C (2007). Continuity of care 2006: What have we learned since 2000 and what are policy imperatives now? Report for the National Co-ordinating Centre for NHS Service Delivery and Organisation R&D.

[B7] Shaw KL, Southwood TR, McDonagh JE (2004). User perspectives of transitional care for adolescents with juvenile idiopathic arthritis. Rheumatology.

[B8] Bain NS, Campbell NC (2000). Treating patients with colorectal cancer in rural and urban areas: a qualitative study of the patients' perspective. Fam Pract.

[B9] Grumbach K, Selby JV, Damberg C, Bindman AB, Quesenberry C, Truman A, Uratsu C (1999). Resolving the gatekeeper conundrum – What patients value in primary care and referrals to specialists. JAMA.

[B10] Jenkinson C, Coulter A, Bruster S, Richards N, Chandola T (2002). Patients' experiences and satisfaction with health care: results of a questionnaire study of specific aspects of care. Qual Saf Health Care.

[B11] Bruster S, Jarman B, Bosanquet N, Weston D, Erens R, Delbanco TL (1994). National Survey of Hospital Patients. BMJ.

[B12] Hruby M, Pantilat SZ, Lo B (2007). How do patients view the role of the primary care physician in inpatient care?. Am J Med.

[B13] Burkey Y, Black M, Reeve H (1997). Patients' views on their discharge from follow up in outpatient clinics: qualitative study. BMJ.

[B14] Nielsen JD, Palshof T, Mainz J, Jensen AB, Olesen F (2003). Randomised controlled trial of a shared care programme for newly referred cancer patients: bridging the gap between general practice and hospital. Qual Saf Health Care.

[B15] Arthur V, Clifford C (2004). Rheumatology: the expectations and preferences of patients for their follow-up monitoring care: a qualitative study to determine the dimensions of patient satisfaction. J Clin Nurs.

[B16] Infante FA, Proudfoot JG, Powell Davies G, Bubner TK, Holton CH, Beilby JJ, Harris MF (2004). How people with chronic illnesses view their care in general practice: a qualitative study. Med J Aust.

[B17] Cleary PD, EdgmanLevitan S (1997). Health care quality – Incorporating consumer perspectives. JAMA.

[B18] Richards T (1999). Patients' priorities – Need to be assessed properly and taken into account. BMJ.

[B19] Sixma HJ, Kerssens JJ, van Campen C, Peters L (1998). Quality of care from the patients' perspective: from theoretical concept to a new measuring instrument. Health Expect.

[B20] Malterud K (2001). Qualitative research: standards, challenges, and guidelines. Lancet.

[B21] Ritchie J, Spencer L, Bryman A, Burgess R (1994). Qualitative data analysis in applied policy research. Analyzing Qualitative Data.

[B22] Peters V, Wester F (2007). How qualitative software may support the qualitative analysis process. Quality & Quantity.

[B23] Pope C, van Royen P, Baker R (2002). Qualitative methods in research on healthcare quality. Qual Saf Health Care.

[B24] Mitchell G, Del Mar C, Francis D (2002). Does primary medical practitioner involvement with a specialist team improve patient outcomes? A systematic review. Br J Gen Pract.

[B25] Suhonen R, Nenonen H, Laukka A, Valimaki M (2005). Patients' informational needs and information received do not correspond in hospital. J Clin Nurs.

[B26] Rosemann T, Wensing M, Rueter G, Szecsenyi J (2006). Referrals from general practice to consultants in Germany: If the GP is the initiator, patients' experiences are more positive. BMC Health Serv Res.

[B27] Bowling A, Culliford L, Smith D, Rowe G, Reeves BC (2008). What do patients really want? Patients' preferences for treatment for angina. Health Expect.

